# Koffeinintoxikation in suizidaler Absicht

**DOI:** 10.1007/s00108-025-01938-w

**Published:** 2025-06-23

**Authors:** Sabrina Uehlein, Reinhard Schneider, Klaus Stahl, Katharina Dechant

**Affiliations:** 1 Medizinische Klinik 2 – Kardiologie, Angiologie, Universitätsklinikum Erlangen, Friedrich-Alexander-Universität Erlangen-Nürnberg, Erlangen, Deutschland; 2 Klinik für Kardiologie und Angiologie, Medizinische Hochschule Hannover, MHH, Carl-Neuberg-Straße 1, 30625 Hannover, Deutschland; 3 Klinik für Gastroenterologie, Hepatologie, Infektiologie und Endokrinologie, Medizinische Hochschule Hannover, MHH, Hannover, Deutschland

**Keywords:** Emesis, Hämodialyse, Polyurie, Laktatazidose, Hypokaliämie, Emesis, Hemodialysis, Polyuria, Lactic acidosis, Hypokalemia

## Abstract

Nach oraler Einnahme von 40 g Koffein in suizidaler Absicht zeigte sich ein 34-jähriger Patient stark symptomatisch mit progredienter Nausea, Emesis und Polyurie. Das Labor des Patienten wies eine Laktatazidose, Rhabdomyolyse, Hypokaliämie und Hypernatriämie auf. Es erfolgte eine symptomatische Therapie sowie eine intravenöse Flüssigkeits- und Elektrolytsubstitution. Mittels Hämodialyse ließ sich eine ausreichende Elimination des Koffeins bewirken. Im weiteren Verlauf wurde der Patient bei schwerer Depression in die Abteilung für Psychiatrie verlegt.

## Abstract (en)

Following oral ingestion of 40 g of caffeine with suicidal intent, a 34-year-old male patient presented with worsening nausea, emesis and polyuria. The patient’s lab results showed lactic acidosis, rhabdomyolysis, hypokalemia and hypernatremia. Symptomatic treatment as well as fluid and electrolyte replacement were initiated. By means of hemodialysis, sufficient elimination of caffeine could be achieved. After stabilization, the patient was transferred to the department of psychiatry and psychotherapy for treatment of his depression.

## Anamnese

Ein 34-jähriger Patient hatte am Vorabend gegen 22 Uhr in suizidaler Intention kumulativ 40 g Koffein in Tablettenform zusammen mit zwei Flaschen alkoholhaltigem Bier eingenommen. Gegen 24 Uhr habe sich eine ausgeprägte Nausea mit konsekutiver Emesis sowie Polyurie entwickelt. Vor der Tabletteneinnahme war eine Abschieds-SMS mit programmierter Zeitverzögerung an die im Ausland wohnhaften Eltern versendet worden, welche nach Erhalt der Nachricht den ortsansässigen Rettungsdienst verständigten. Bei Eintreffen des Rettungsdiensts und der Polizei in der Wohnung des Patienten am darauffolgenden Morgen fanden diese den Patienten wach, kontaktfähig, stark exsikkiert sowie tachykard und tachypnoisch vor. Vorbekannt war eine schwere Depression mit bereits zweimalig erfolgtem medikamentösem Suizidversuch. Während des Transports in die Notaufnahme blieb der Zustand des Patienten unverändert, dort traf er gegen Mittag ein.

## Befund

In der klinischen Untersuchung präsentierte sich der Patient tachypnoisch in einem deutlich reduzierten Allgemeinzustand mit Kaltschweißigkeit, Exsikkose und blassem Hautkolorit bei normalem Ernährungszustand.

Das Aufnahme-EKG erbrachte einen normofrequenten Sinusrhythmus. Im weiteren Monitoring fiel eine gehäufte monomorphe ventrikuläre Extrasystolie auf.

In der initialen venösen Blutgasanalyse in der Notaufnahme zeigte sich eine respiratorisch kompensierte Additionsazidose mit vergrößerter Anionenlücke von 25,7 mmol/l bei Laktatazidose mit einem Laktat von 10,1 mmol/l, einer Hypokaliämie von 2,6 mmol/l sowie einer Hypernatriämie von 152 mmol/l (Tab. 1).

**Tab. 1:** Verlauf der Blutgasanalysen

Verlauf der Blutgasanalysen		0 h	+6,5 h	+12,5 h	+19,5 h	+25,5 h	+34,5 h	+45,5 h
			*Unter HD/Genius-HD/CiCa*					
Art		*Venös*	*Arteriell*					
pH		7,40	7,69	7,49	7,54	7,50	7,49	7,53
pCO_2_	[mmHg]	28,9	14,7	24,7	27,4	28,2	31,1	31,2
sO_2_	[%]	54,4	99,3	99,0	98,9	98,4	98,0	98,2
Kalium	[mmol/l]	2,6	3,7	4,1	4,6	4,1	4,4	4,3
Natrium	[mmol/l]	152	144	141	140	141	137	137
Laktat	[mmol/l]	10	3,0	1,2	1,0	0,7	0,6	0,9
Glukose	[mg/dl]	208	126	114	121	108	107	122
Anionenlücke	[mmol/l]	25,7	12,1	6,7	4,7	6,5	6,1	5,9
Osmolalität	[mmol/kg]	315	295	289	288	288	280	282
Basenexzess	[mmol/l]	−6,7	−2,4	−2,7	0,6	−0,9	0,7	3,2
Standard $\mathrm{HC}\mathrm{O}_{3}^{-}$	[mmol/l]	19,2	24,8	22,2	25,9	24,5	25,6	27,9

Laborchemisch fielen eine deutlich erhöhte Kreatin-Kinase mit einem Wert von 3661 U/l, eine Hypophosphatämie von 1,1 mg/dl sowie eine Serumkoffeinkonzentration von 91,9 mg/l auf. Weitere Laborwerte finden sich kumulativ in Tab. 2.

**Tab. 2:** Laborwerte kumulativ

**Blutbild**	**Wert**	**Normwert**	**Einheit**	**Serum**	**Wert**	**Normwert**	**Einheit**
Leukozyten	**19,1**	*4–10*	x10^3^/μl	Natrium	150	*135–145*	mmol/l
Hämoglobin	**13,2**	*13,5–17,5*	g/dl	Kreatinin	1,16	*0,67–1,17*	mg/dl
Thrombozyten	250	*140–350*	x10^3^/μl	GFR	**82**	*90–120*	ml/min/1,72 m^2^
Erythrozyten	4,5	*4,4–5,8*	x10^6^/μl	Harnstoff	**33**	*17–43*	mg/dl
Hämatokrit	36,2	*39–51*	%	Kalzium	2,57	*2,2–2,65*	mmol/l
MCH	29,6	*27–33*	pg	Osmolalität Gefrierpunktserniedrigung	**316**	*280–300*	mosmol/kg
MCV	81,2	*81–98*	fl	Osmolalität rechner.	**318**	*280–300*	mosmol/kg
MCHC	36,5	*32–36*	g/dl	Osmotische Lücke	−2	*<* *7*	mosmol/kg
RDW	13,2	*11,6–16,2*	%	CRP	**27,4**	*<* *5*	mg/dl
MPV	8,9	*7,8–11*	fl	Prokalzitonin	0,79	*<* *0,5*	ng/ml
**Diff.-Blutbild maschinell**	**Wert**	**Normwert**	**Einheit**	Albumin	46,3	*35–55*	g/l
Granulozyten absolut	**16,20**	*1,8–9,0*	x10^3^/μl	AST	**202**	*<* *50*	U/l
Granulozyten in *%*	**85,00**	*41–74*	%	ALT	20	*<* *50*	U/l
Lymphozyten absolut	1,14	*1–3*	x10^3^/μl	LDH	**503**	*<* *250*	U/l
Lymphozyten in %	6,00	*18–46*	%	Gamma-GT	26	*<* *29*	U/l
Eosinophile absolut	0,00	*0–0,6*	x10^3^/μl	Lipase	**15**	*<* *60*	U/l
Eosinophile in %	0,00	*0–7*	%	Bilirubin gesamt	0,4	*<* *1,1*	mg/dl
Monozyten absolut	1,70	*0,2–1*	x10^3^/μl	Triglyzeride	45	*50–200*	mg/dl
Monozyten in %	8,90	*4–15*	%	HbA1c	5,1	*4,4–6,0*	%
Erythrozytenverteil.	37,20	*37–46*	fl	HbA1c	31,8	*25–42*	mmol/mol
Große Thrombozyten	**15,60**	*17,5–42*	%	TSH	3,57	*0,2–4,0*	mlU/l
Thrombozytenverteil.	**9,50**	*10–16*	fl	Troponin	**5,6**	*<19,8*	pg/ml
Basophile absolut	0,02	*0–0,007*	x10^3^/μl	CK	**3661**	*292*	U/l
Basophile in %	0,10	*0–1,2*	%	CK-MB	1,7	*<6,0*	%
Anteil makrozytärer Erythrozyten	3,80	*0–5*	%	Magnesium	**0,7**	*0,7–1,1*	mmol/l
Erythrozyten Verteilungsbreite	**12,6**	*12–14,3*	%	Kalzium	2,57	*2,2–2,65*	mmol/l
Mittleres Thrombozytenvol.	**8,80**	*9–12*	fl	Anorgan. Phosphat	**1,1**	*2,5–4,5*	mg/dl
Normoblasten	0,00	*<* *1*	%	Anorgan. Phosphat	**0,36**	*0,81–1,45*	mmol/l
Unreife Granulozyten	1,00	*0,0–0,6*	%	Ca-Phosphat-Produkt	0,93	*<* *4,4*	mmol^2^/l^2^
**Gerinnung**	**Wert**	**Normwert**	**Einheit**	Glukose	218	*70–110*	mg/dl
Quick	100	> *70*	%	**Mikrobiologie**	**Wert**	**Normwert**	**Einheit**
INR	1,0	*0,85–1,15*	–	SARS-CoV-2-mRNA	Neg.	*Neg.*	–
aPTT	25,6	*23,6–34,8*	s	Blutkulturen	Neg.	*Neg.*	–
**Toxikolog. Screening**	**Wert**	**Normwert**	**Einheit**	**Toxikolog. Screening**	**Wert**	**Normwert**	**Einheit**
Theophyllin	**6,5**	*10–20*	μg/ml	Ethanol	< 0,1	–	‰
Barbiturate	Neg.	*<* *0,5*	μg/ml	Acetaminophen	**<** **10**	*10–30*	μg/ml
Benzodiazepine	Neg.	*<* *0,003*	μg/ml	Trizykl. Antidepressiva	Neg.	*<* *300*	ng/ml

## Therapie und Verlauf

**Abb. 1:**
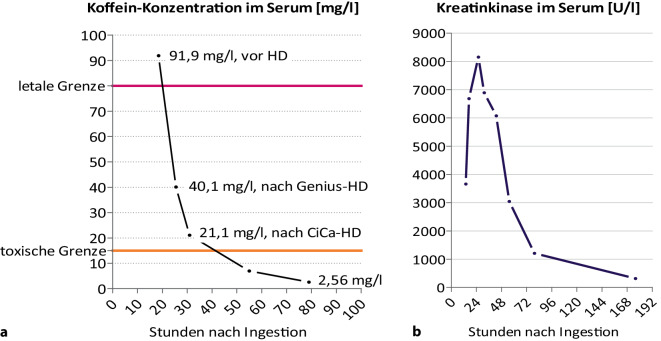
**a** Gemessene Koffeinkonzentration im Serum mittels HPLC und **b** gemessene Kreatinkinasekonzentration im zeitlichen Verlauf

In der Notaufnahme wurde mit einer intravenösen Flüssigkeits- und Elektrolytsubstitution, einer antiemetischen Therapie mit sedierender Wirkung mittels Dimenhydrinat und mit Pantoprazol begonnen. Anschließend erfolgte die Verlegung auf die internistische Intensivstation zur weiteren Überwachung und zum Beginn einer Hämodialyse gemäß der Rücksprache mit der Giftnotrufzentrale in München. Zur Elimination des Koffeins wurde gemäß der Empfehlung der Kollegen für Nephrologie zunächst eine Genius®-Hämodialyse (Fresenius Medical Care, Bad Homburg, Deutschland) über sechs Stunden sowie eine anschließende CiCa®-SLED (Fresenius Medical Care, Bad Homburg, Deutschland) mit CytoSorb®-Filter (CytoSorbents Corp., NJ, USA) durchgeführt. Unter der jeweils sechsstündigen Hämodialyse ließ sich eine Halbierung der Koffeinserumkonzentration erzielen (Abb. 1a).

Die symptomatische, antiemetische Therapie wurde bei therapierefraktärer Emesis nach Verlegung auf die internistische Intensivstation um Ondansetron und Metoclopramid erweitert. Unter Ausdosierung der Medikation besserte sich die Symptomatik rasch. Bei klinisch und radiologisch fehlenden Hinweisen auf emesisbedingte Komplikationen, wie ein Boerhaave-Syndrom oder Mallory-Weiss-Syndrom, wurde ein Kostaufbau am Folgetag begonnen und auf eine Ösophagogastroduodenoskopie verzichtet. Im weiteren Verlauf konnte die Substitutionstherapie mittels Glyzeroldihydrogenphosphat und Kaliumchlorid beendet werden. Während des intensivstationären Aufenthalts wurden eine Harnalkalisierung mittels Kaliumnatriumhydrogencitrat sowie eine Flüssigkeitssubstitution bei initial noch weiter steigenden Kreatin-Kinase-Werten i. S. einer Rhabdomyolyse vorgenommen. Auch hier zeigten sich im Verlauf die Werte rückläufig (Abb. 1b). Bei erhöhten Körpertemperaturen, steigenden laborchemischen Entzündungsparametern und anhaltenden Schluckbeschwerden wurden eine kalkulierte antibiotische Therapie mittels Ampicillin/Sulbactam sowie eine erneute Röntgenaufnahme des Thorax z. A. emesisbedingter Komplikationen, wie einer Aspirationspneumonie oder Ösophagusruptur, durchgeführt. Diese war unauffällig.

**Abb. 2:**
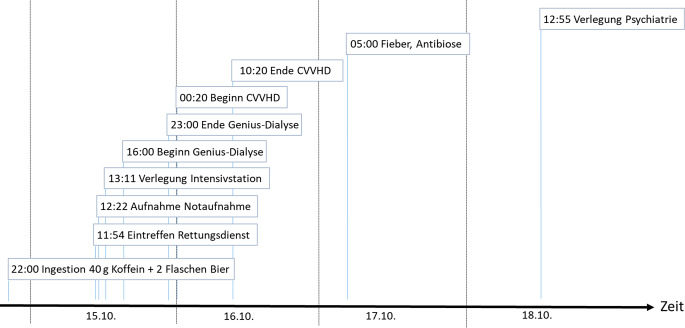
Zeitachse zum klinischen Verlauf des Patienten

Nach ausreichender metabolischer Stabilisierung (Tab. 1) ohne Rebound- oder Rhythmusereignisse, regredienten Kreatin-Kinase-Werten (Abb. 1b) und normalisierten Koffeinkonzentrationen (Abb. 1a) erfolgte eine Verlegung in die Abteilung für Psychiatrie zum Ausschluss einer hirnorganischen Genese sowie zum Beginn eines multimodalen Behandlungskonzepts bei schwerer Depression. In Abb. 2 findet sich der klinische Verlauf als Zeitachse dargestellt.

## Diskussion

In unserem vorgestellten Fall entsprach die eingenommene Koffeinmenge von 40 g umgerechnet ca. 471 Filterkaffeetassen à 250 ml mit einem durchschnittlichen Koffeingehalt von 85 mg pro Tasse.

**Abb. 3:**
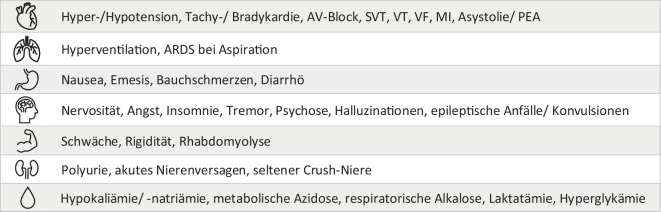
Symptome bei Koffeinintoxikation nach Organsystemen sortiert (*AV* atrioventrikulär, *SVT* supraventrikuläre Tachykardie, *VT* ventrikuläre Tachykardie, *VF* Kammerflimmern, *MI* Myokardischämie, *PEA* pulslose elektrische Aktivität, *ARDS* akutes respiratorisches Distress-Syndrom)

Symptome einer Intoxikation (Abb. 3) können ab einer Dosis von ca. ein bis zwei Gramm Koffein bzw. Serumkonzentrationen über 15 mg/l auftreten. Als letale Dosis bzw. letale Serumkonzentrationen werden in der Literatur Dosen ab fünf bis zehn Gramm bzw. 100 bis 200 mg/kg sowie Blutkonzentrationen > 80 mg/l angegeben [1, 3, 7, 9].

Die intestinale Resorption von oral eingenommenem Koffein erfolgt bei einer Bioverfügbarkeit von ca. 99 % [9] innerhalb von ca. 30 min [2], der Plasmapeak wird nach etwa einer Stunde erreicht [1]. Der Abbau findet in der Leber über das Cytochrom-P450-System durch das CYP1A2-Enzym in die Metaboliten statt und wird renal eliminiert [9]. Die Eliminationshalbwertszeit kann zwischen drei und sechs Stunden betragen und wird durch verschiedene Faktoren [1, 2, 5, 9], u. a. durch die eingenommene Koffeinmenge, beeinflusst. Bei Koffeinintoxikationen kann sich die Eliminationshalbwertszeit mit der eingenommenen Dosis verlängern, u. a. aufgrund der Abbauprodukte von Koffein, welche ebenfalls über das CYP1A2-Enzym metabolisiert werden, z. B. Theophyllin [9].

In Anbetracht der o. g. Pharmakologie, der eingenommenen Dosis von 40 g sowie der potenziell letalen Serumkonzentration von 91,9 mg/l wird vermutlich ein Großteil des Koffeins aufgrund der starken Emesis bei beginnender Intoxikation nicht resorbiert worden sein. Nichtsdestotrotz wird der Plasmapeak in der Häuslichkeit deutlich höher als der 12 h nach oraler Aufnahme gemessene Wert von 91,9 mg/l in der Notaufnahme gewesen sein.

In Anbetracht des 12-stündigen Intervalls zwischen intestinaler Resorption und Vorstellung in der Notaufnahme sowie der anhaltend ausgeprägten Emesis entschieden wir uns gegen die Gabe von Aktivkohle sowie gegen eine Magenspülung, wie es in anderen Fallberichten bei Koffeinintoxikationen durchgeführt worden ist [6–8].

Unter der Koffeinintoxikation kam es während des stationären Aufenthalts zu keinen Rebound-Ereignissen, tachykarden (supra-)ventrikulären Herzrhythmusstörungen oder epileptischen Anfällen, worüber bei schweren Intoxikationen berichtet worden war [2, 4–6, 8].

Als therapeutisch wirksam bei schweren Koffeinintoxikationen wurde neben der Hämodialyse [1, 2, 5] ebenfalls die intravenöse Gabe von Lipidemulsionen beschrieben [4, 6–8].

## Fazit für die Praxis

Im Falle von Emesis, Tachypnoe, Polyurie sowie ggf. kardialen und neurologischen Auffälligkeiten sowie bei Nachweis einer Laktatazidose, Hypokaliämie, Hyperkaliämie mit erhöhter Kreatin-Kinase sollte an eine Koffeinintoxikation gedacht werden. Eine offizielle Therapieempfehlung zu Koffeinintoxikationen besteht aktuell nicht. Jedoch sollte frühzeitig eine Hämodialyse zur Koffeinelimination in Betracht gezogen werden, insbesondere bei unklarer oder potenziell letaler Ingestionsmenge.
